# Return to normal activity after abdominal surgery: a pre-planned secondary analysis of a randomised controlled trial across seven low- and middle-income countries

**DOI:** 10.1186/s12893-025-03079-1

**Published:** 2025-08-29

**Authors:** Omar Omar, Sivesh Kathir Kamarajah

**Affiliations:** NIHR Global Health Research Unit on Global Surgery, Institute of Applied Health Research, University of Birmingham, Birmingham, UK

**Keywords:** Abdominal surgery, Return to normal activity, Return to work

## Abstract

**Background:**

Recovery after major surgery is a key priority identified by patients, communities and policymakers in low- and middle-income countries (LMICs), with important societal and financial implications. With global burden of surgical diseases rising, little is known about how well patients return to normal activities after surgery in these settings. This study aimed to describe patterns of return to normal activity after major abdominal surgery and identify associated factors in LMICs.

**Methods:**

This was a pre-planned analysis of a cluster-randomised randomised trial testing routine sterile glove and instrument change at the time of abdominal wound closure to prevent surgical site infection in seven LMICs (India, Mexico, Rwanda, Benin, South Africa, Nigeria, Ghana). 961 patients were excluded because of incomplete missing primary outcome. The primary outcome measure was a patients self-reported full return to their normal activities at 30 days after surgery. Factors associated with return to normal activities within 30-days of surgery was explored using a Bayesian mixed-effects logistic regression model. Sensitivity analyses were performed accounting for missing data.

**Results:**

12,340 patients across 81 centres were included. Overall, 65.3% (8064/12340) patients had returned to normal activity by 30-days after surgery. Patients undergoing surgery for benign than cancer surgery (67.0% vs. 59.7%), minor compared to major surgery (71.0% vs. 63.5%), and non-midline compared to midline (74.9% vs. 58.7%) had higher rates of return to normal activities within 30-days from abdominal surgery. In an adjusted model, factors associated with return to normal activities are benign surgery (OR: 0.61, 95% CI: 0.53–0.71), minor surgery (OR: 0.56, 95% CI: 0.49–0.64), and non-midline operations (OR: 1.57, 95% CI: 1.41–1.75). When accounting for missing data, consistent findings were observed.

**Conclusions:**

With rising need for surgical care and non-communicable disease globally, this study highlights the groups of patients at critical need for improving return to normal activity or recovery after surgery in LMICs. Improving access and implementation of rehabilitation pathways, aligned to the World Health Organisation, may be crucial to improve financial risk protection to patient and reduce productivity loss to the economy.

**Supplementary Information:**

The online version contains supplementary material available at 10.1186/s12893-025-03079-1.

## Introduction

Each year, 313 million patients undergo surgery globally each year for a variety of conditions such as osteoarthritis, cancer and congenital disease [1, 2]. These conditions account for 28 − 32% of the global burden of disease. It is expected that the global burden of surgical disease is set to increase over the coming decade [3, 4]. The Lancet Commission on Global Surgery 2030 have highlighted the shortage of essential, safe and affordable surgical procedures to more than half of the world’s population [4]. This shortfall has a negative impact on patient morbidity, mortality and long-term functioning. This led to calls for increasing investment to strengthen surgical systems globally. However, an omitted area from this commission is improving recovery or rehabilitation to normal activity after surgery. Efforts to integrate rehabilitation into clinical care pathways have been identified an urgent priority by the World Health Organisation, in efforts to achieving universal health coverage [5, 6]. 

Improving recovery after surgery is crucial to reduce risk of long-term disability. The ability to return to normal activities can have a huge impact on the patients personal circumstances, psychological wellbeing as well as economic situation [7]. The ability to return to normal activities may affect the patients ability to work, and care for those around them as well as affecting others ability to work conduct their own activities due to caring for the patient. The economic and societal burden, tied in with the personal impact on not being able to return to normal activities shows just how important this issue is. In addition, early mobilisation has been reported to reduce risk of complications in patients, particularly after cancer surgery, increasing likelihood of returning to normal activities [8]. 

At present, the evidence around the scale of return to normal activities or recovery from surgery performed in low- and middle-income countries are lacking. Therefore, this secondary pre-planned analysis aims to assess the rates of return to normal activities in patients undergoing abdominal surgery, and the factors which may hinder this, so that progress can be made to help improve outcomes and reduce this economic and societal burden.

## Methods

### Study design

This study was a pre-planned secondary analysis of the ChEETAh trial across seven LMICs. ChEETAh was a pragmatic, cluster randomised controlled trial comparing the effectiveness of routine change of gloves and instruments before wound closure for the whole scrub team compared to usual care [9]. Final approval for this secondary analysis was given by the ChEETAh trial management group. The trial was approved by the University of Birmingham Research Ethics Committee (ERN_ 19–0719). Ethics and regulatory approvals in each participating country were sought in line with regional or national regulations, or both. The full protocol and results of the trial are reported elsewhere [9]. 

### Inclusion and exclusion criteria

ChEETAh was delivered across 81 hospitals in seven countries (Benin, Ghana, India, Mexico, Nigeria, Rwanda, and South Africa) between June 24, 2020, and March 31, 2022. The trial included both adult and paediatric patients undergoing major abdominal surgery with a planned skin incision of 5 cm or greater. Patients predicted to have a clean-contaminated, or contaminated or dirty procedure were included, regardless of urgency of procedure (i.e., elective or emergency). The trial protocol required follow-up up to 30-days after surgery. If follow-up was not possible at 30-days after surgery, then patients were followed-up soon after this as possible. Follow-up could be conducted either in-person or by telephone; the latter was implemented during final phase of trial recruitment, where many participating countries were affected by the SARS-CoV-2 pandemic. In this analysis we excluded patients that did not undergo 30-day follow-up or that were randomised but then did not undergo surgery as they could not be assessed for the primary outcome measures.

### Outcomes

The primary outcome measure is return to normal activity within 30-days following major abdominal surgery. On 30-day follow-up, all patients received a questionnaire via an in-person or telephone asking whether they had returned to normal activities following surgery. The assessment was done by the research nurses involved in the main clinical trial, at the same point assessment of surgical site infection was made. Since there is no standardised questionnaire for assessing return to normal activity, we made a template questionnaire for use in this study.

### Covariables

For this study, patient-level and operative-level variables were used. Patient-level factors used were age, sex (i.e., male or female), presence of diabetes or HIV, and smoking status. Data on American Society of Anaesthesiologist Physical Status Classification System (ASA Physical status) were included [10]. Operative-level factors were urgency of surgery (i.e., elective or emergency), indication (i.e., benign, malignant, obstetric, and trauma), grade of surgery, use of the WHO checklist, prophylactic antibiotics, surgical approach and contamination. Grade of surgery was defined as minor, intermediate and major according to an internationally recognised definition from the National Institute of Clinical Excellence [11]. A list of surgery types corresponding to the grade have been previously developed and validated, which we mapped them on to. At 30 days, data was collected on whether there was SSI at time of discharge, whether a re-operation or unplanned wound opening was required within 30 days, readmission to hospital and whether there was a return to normal activities within 30 days of surgery.

### Sample size calculation

In the CHEETAH trial, a minimum of 6400 participants per group (12800 total) were required to detect a 25% relative reduction in surgical site infection rate. Assuming a return to normal activity rate of about 65% as observed in the main trial and 95% level of confidence, a sample size of 12,000 equates to a margin of error of approximately 0.85%.

### Statistical analyses

**Fig. 1:**
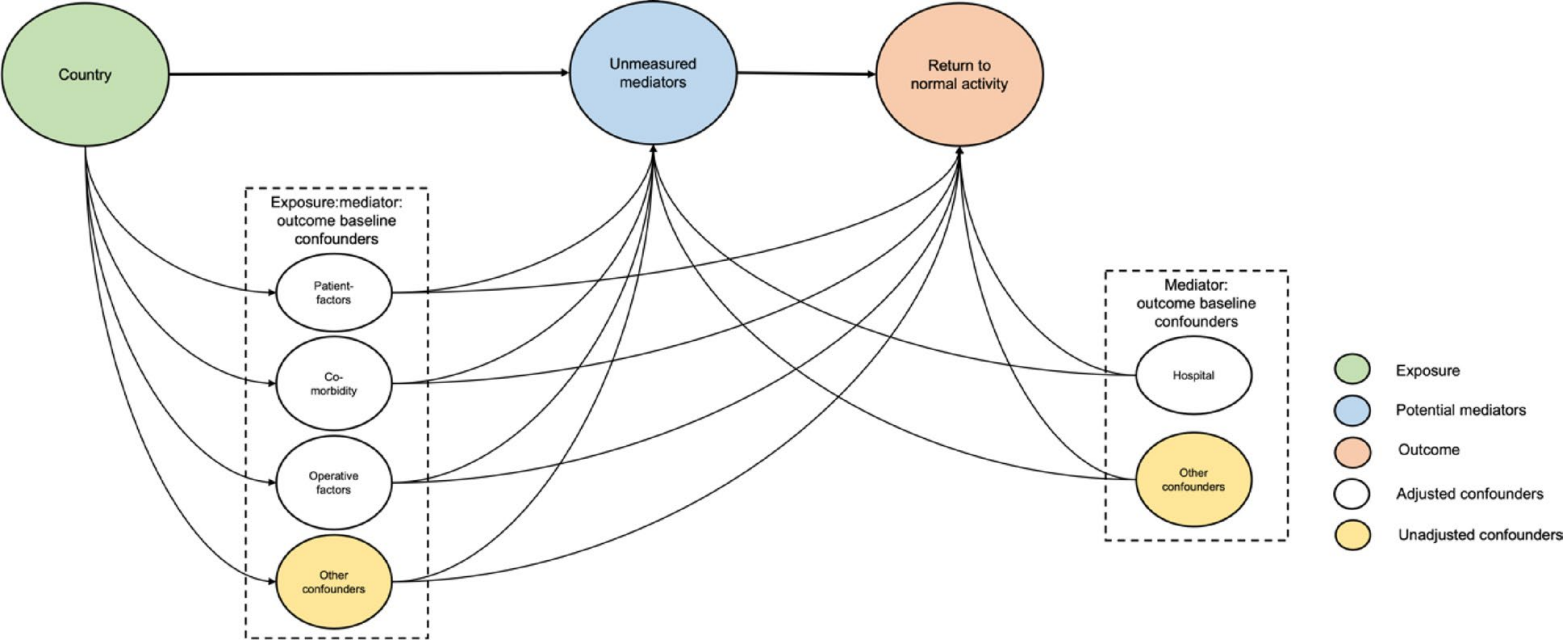
Direct acyclic graph demonstrating relationship between country and return to normal activity within 30-days from surgery

Descriptive data are presented using frequencies and percentages. The Chi-squared test of independence was used to compare groups of categorical data. A Bayesian multivariable random effects logistic regression model was used to evaluate the association of variables and the return to normal activities following abdominal surgery, which were then summarised using the odds ratios (OR) and 95% credible intervals (CI). Hospital was included as a random effect in the adjusted model. The student *t* distribution t (3,0,2.5) was assumed for the intercept and weakly informative priors were assumed for all the coefficients in the model (normal distribution (0,10)) and standard deviation for random effects random effect (Half Cauchy distribution centred at 0 and scaled to 5). The model included clinically relevant patient-level factors (i.e. age, sex and ASA grade of the patient) and operative-level factors (i.e. urgency, indication for surgery, contamination, surgical approach, grade of surgery, whether the WHO checklist was used and whether change of gloves/instruments was done prior to wound closure). A direct acyclic graph to explain the relationship are presented in Fig. 1. A complete-case analysis was pre-planned if missing data were both missing at random and in a low number of samples (< 5%) [12]. In the pre-study protocol, we planned to impute missing data using multiple imputation by chained equations based on a missing at random or missing completely at random assumption if data missingness was more than 5%. To assess the impact of missing data, sensitivity analysis was performed using multiple imputation by chained equations where missing values were assumed to be missing at random. Twenty imputed datasets were created by replacing missing values with simulated values from a set of imputation [13]. Bayesian multivariable random effects logistic regression model was performed on each imputed dataset and the imputation-specific coefficients were combined using Rubin’s rules. Planned subgroup analyses were performed for age groups (adults vs. children) and gender (male vs. female). The following first order interactions were explored: Grade and timing of surgery; timing of surgery and surgical approach and; wound contamination and surgical approach. Analyses were performed in RStudio version 2023.03.0 + 386 packages finalfit, tidyverse, and BRMS.

### Role of the funding source

The sponsor of the study had no role in study design, data collection, data analysis, data interpretation, or writing of the report. The corresponding author had full access to all the data in the study and had final responsibility for the decision to submit for publication.

## Results

### Baseline characteristics

**Fig. 2:**
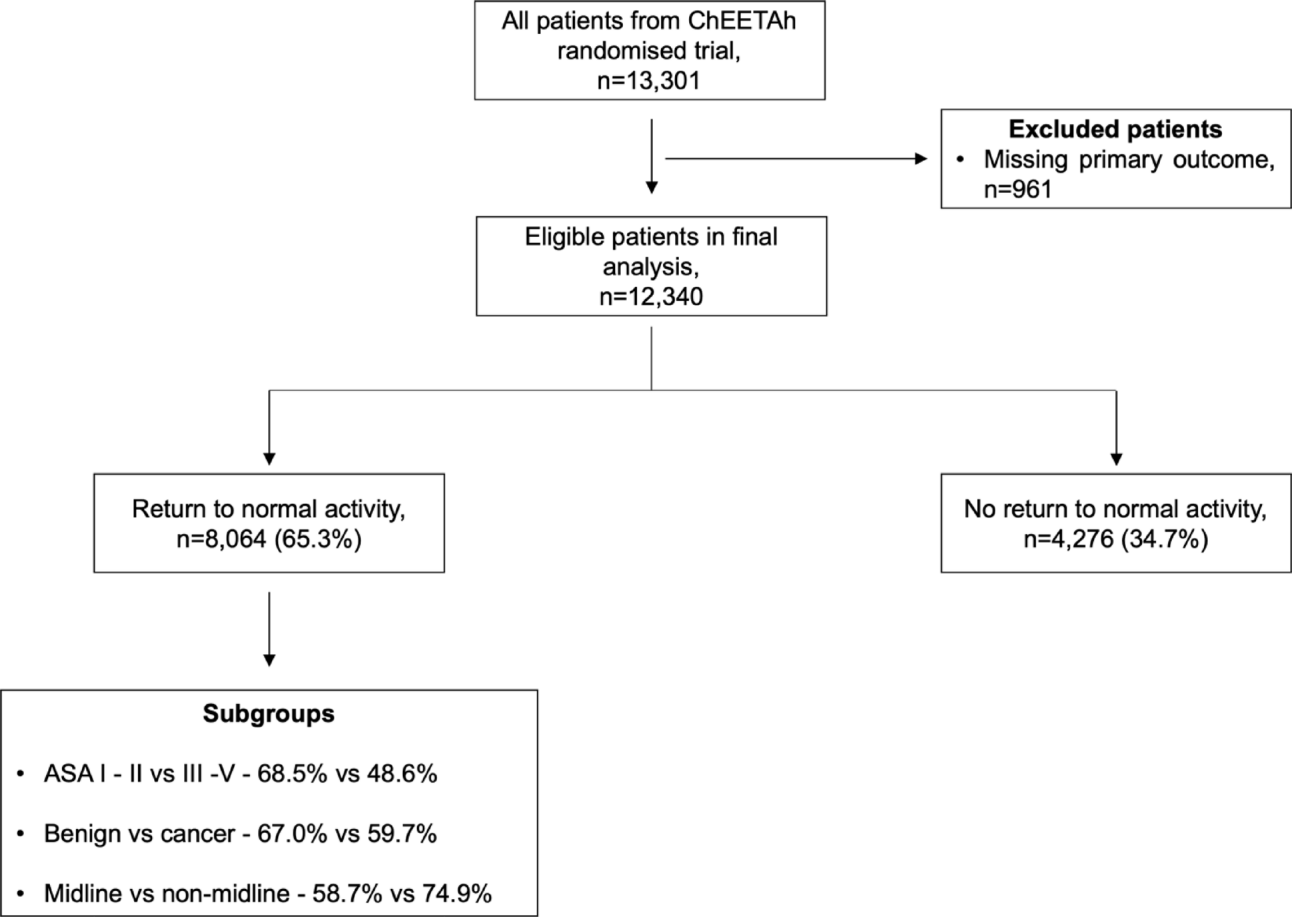
Flow chart of included patients in this secondary analysis

From 13,301 patients, this secondary pre-planned analysis excluded 961 patients (7.2%) because data on the primary outcome was not available (Table S1 & S2). This analysis included 12,340 patients (Fig. 2), of which 87.3% (*n* = 10,771) were adults and 55.9% (*n* = 6,893) were female. 6,478 (52.5%) patients were emergency and 5,862 (47.5%) were elective operations. Of the included patients, 7,280 (59.0%) operations were undertaken via an open midline approach. A summary of the baseline patient- and operative-level characteristics are presented in Tables 1 and 2, respectively.

**Table 1:** Baseline patient-level characteristics of patients with and without return to normal activities at 30-days from surgery

	No (*n* = 4276)	Yes (*n* = 8064)	*p*-value	Total (*n* = 12340)
Age group				
Adult	3781 (35.1)	6990 (64.9)		10,771 (87.3)
Child	495 (31.5)	1074 (68.5)	0.006	1569 (12.7)
Sex				
Male	2069 (38.0)	3378 (62.0)		5447 (44.1)
Female	2207 (32.0)	4686 (68.0)	< 0.001	6893 (55.9)
ASA Grade				
Grade I – II	3278 (31.5)	7121 (68.5)		10,399 (84.3)
Grade III-V	998 (51.4)	943 (48.6)	< 0.001	1941 (15.7)
Diabetes				
No	4015 (34.7)	7562 (65.3)		11,577 (93.8)
Yes	261 (34.2)	502 (65.8)	0.820	763 (6.2)
Smoking				
Never smoked	3778 (33.9)	7361 (66.1)		11,139 (90.3)
Ex-smoker	283 (45.4)	341 (54.6)		624 (5.1)
Current smoker	215 (37.3)	362 (62.7)	< 0.001	577 (4.7)
HIV status				
Negative	2516 (35.0)	4669 (65.0)		7185 (58.2)
Positive	80 (31.6)	173 (68.4)		253 (2.1)
Not known	1680 (34.3)	3222 (65.7)	0.414	4902 (39.7)
Timing of surgery				
Elective	1914 (32.7)	3948 (67.3)		5862 (47.5)
Emergency	2362 (36.5)	4116 (63.5)	< 0.001	6478 (52.5)
Follow up method				
Clinical notes/charts	101 (58.0)	73 (42.0)		174 (1.4)
In-person, community	14 (36.8)	24 (63.2)		38 (0.3)
In-person, hospital	939 (46.2)	1092 (53.8)		2031 (16.5)
Phone call	3222 (31.9)	6875 (68.1)	< 0.001	10,097 (81.8)

**Table 2:** Baseline operative-level characteristics of patients with and without return to normal activities at 30-days from surgery

	No (*n* = 4276)	Yes (*n* = 8064)	*p*-value	Total (*n* = 12340)
Indication				
Benign	3247 (33.0)	6592 (67.0)		9839 (79.7)
Malignant	776 (40.3)	1150 (59.7)		1926 (15.6)
Trauma	253 (44.0)	322 (56.0)	< 0.001	575 (4.7)
*Missing*	0	0		0
Surgery type				
Appendicectomy	465 (23.6)	1504 (76.4)	< 0.001	1969 (16.0)
Colorectal	597 (44.5)	745 (55.5)		1342 (10.9)
Gynaecology	812 (26.9)	2210 (73.1)		3022 (24.5)
Hepatobiliary	242 (27.8)	629 (72.2)		871 (7.1)
Laparotomy	670 (43.2)	881 (56.8)		1551 (12.6)
Small bowel	424 (49.4)	435 (50.6)		859 (7.0)
Upper GI	261 (40.8)	378 (59.2)		639 (5.2)
Urology	215 (32.2)	453 (67.8)		668 (5.4)
Other	590 (41.6)	829 (58.4)		1419 (11.5)
*Missing*	0	0		0
Grade of surgery				
Minor	869 (29.0)	2130 (71.0)		2999 (24.3)
Major	3407 (36.5)	5932 (63.5)	< 0.001	9339 (75.7)
*Missing*	0	2		2
WHO checklist				
No	170 (37.8)	280 (62.2)		450 (3.6)
Yes	4106 (34.5)	7784 (65.5)	0.171	11,890 (96.4)
*Missing*	0	0		0
Pulse oximetry				
No	12 (32.4)	25 (67.6)		37 (0.3)
Yes	4264 (34.7)	8039 (65.3)	0.912	12,303 (99.7)
*Missing*	0	0		0
Prophylactic antibiotics				
No	51 (25.4)	150 (74.6)		201 (1.6)
Yes	4225 (34.8)	7914 (65.2)	0.007	12,139 (98.4)
*Missing*	0	0		0
Surgical approach				
Midline	3007 (41.3)	4273 (58.7)		7280 (59.0)
Non-Midline	1269 (25.1)	3791 (74.9)	< 0.001	5060 (41.0)
*Missing*	0	0		0
Contamination				
Clean + Clean-contaminated	2315 (29.9)	5419 (70.1)		7734 (62.7)
Contaminated	1005 (40.1)	1503 (59.9)		2508 (20.3)
Dirty	956 (45.6)	1142 (54.4)	< 0.001	2098 (17.0)
Missing	0	0		0
Change of gloves and/or instruments				
No	2222 (33.3)	4443 (66.7)		6665 (54.0)
Yes	2054 (36.2)	3621 (63.8)	0.001	5675 (46.0)
*Missing*	0	0		0

### Return to normal activities

Of 12,340 patients, only 65.3% (*n* = 8,064/12,340) reported a complete return to normal activity within 30-days following abdominal surgery in LMICs. Return to normal activities were higher in patients with ASA grade I - II (68.5% vs. 48.6%, *p* < 0.001); compared to ASA grade III - V), never smokers (66.1% vs. 54.6% vs. 62.7%, *p* < 0.001, compared to current and ex-smokers) and those undergoing elective surgery (67.3% vs. 63.5%, *p* < 0.001; compared to emergency surgery). (Table 1) Patients undergoing surgery for benign than cancer surgery (67.0% vs. 59.7%, *p* < 0.001), minor compared to major surgery (71.0% vs. 63.5%, *p* < 0.001), and non-midline compared to midline (74.9% vs. 58.7%, *p* < 0.001) had higher rates of return to normal activities within 30-days from abdominal surgery compared to malignant, major surgery and midline operations, respectively (Table 2). Return to normal activity was higher in patients without surgical site infection (70.5% vs. 38.7%), reoperation within 30-days (66.9% vs. 19.8%) and readmission within 30-days infection (66.8% vs. 26.1%) (Table 3).

**Table 3:** Association between return to normal activity and postoperative outcomes within 30-days from abdominal surgery in low- and middle-income countries

	No (*n* = 4276)	Yes (*n* = 8064)
Surgical site infection		
No	3053 (29.5)	7293 (70.5)
Yes	1223 (61.3)	771 (38.7)
Missing	0	0
Reoperation within 30 days		
No	3956 (33.1)	7985 (66.9)
Yes	320 (80.2)	79 (19.8)
Missing	0	0
Wound open within 30 days		
No	3509 (31.2)	7755 (68.8)
Yes	766 (71.3)	309 (28.7)
Missing	1	0
Readmission within 30 days		
No	3953 (33.2)	7950 (66.8)
Yes	323 (73.9)	114 (26.1)
Missing	0	0

**Fig. 3:**
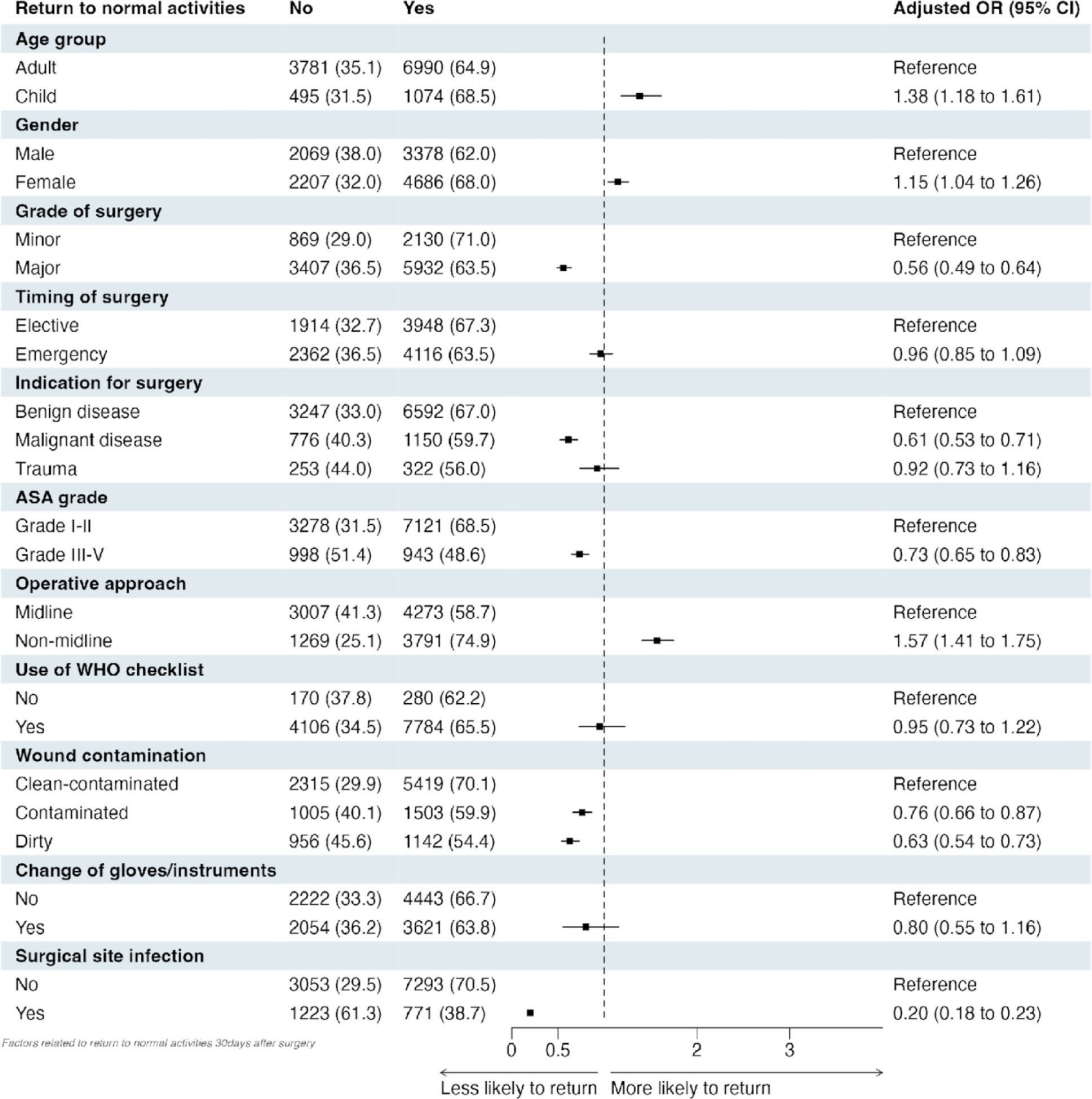
Adjusted Bayesian multivariable regression analysis for patients undergoing abdominal surgery for the outcome of return to normal activity within 30-days from surgery

On adjusted analysis, accounting for patient- and operative-level factors, patients who are female (OR: 1.15, 95% CI: 1.04–1.26) and those undergoing non-midline operations (OR: 1.57, 95% CI: 1.41–1.75) were more likely to return to normal activities. However, patients undergoing surgery with ASA grade III - V (OR: 0.73, 95% CI: 0.65–0.83), major procedures (OR: 0.56, 95% CI: 0.49–0.64), malignant indication (OR: 0.61, 95% CI: 0.53–0.71), contaminated (OR: 0.76, 95% CI: 0.66–0.87) or dirty (OR: 0.63, 95% CI: 0.54–0.73) were less likely to return to normal activities within 30-days of surgery (Fig. 3). Patients who have a surgical site infection within 30-days of surgery were also less likely to return to normal activities. There was no impact on timing of surgery, those undergoing trauma surgery or use of WHO checklist on the return to normal activities. When accounting for missing data, consistent findings were observed (Figure S1). Interaction analysis adjusting for all the different patient- and operative factors identified patients undergoing midline surgery under emergency (OR: 0.55, 95% CI: 0.47–0.64) or with contaminated or dirty wound (OR: 0.35, 95% CI: 0.30–0.40) are least likely to return to normal activity (Figure S2).

### Sensitivity analysis

Two pre-planned sensitivity analyses were performed by age groups (i.e., adults vs. children) and patient gender (i.e., male vs. female). In the children, only those undergoing major surgery, and contaminated or dirty wounds were less likely to return to normal activities (Table S3). This contrasts the sensitivity analyses for adults only where findings were consistent with the overall analysis (Table S4). In contrast, consistent findings were identified with the main analysis for both males and females only sensitivity analysis. The full adjusted model is presented in Table S5 and S6.

## Discussion

To the best of our knowledge, this secondary pre-planned analysis, including over 12,000 patients, is the first to describe return to normal activity after abdominal surgery across seven low- and middle-income countries. The study identified several key findings. First, up to one-in-three patients do not return to normal activity within 30 days after abdominal surgery. Second, particular groups of patients who are comorbid, undergoing major or emergency surgery are at higher risk of not returning back to normal activity levels. These findings highlight the urgent need for investment in improving post-surgery recovery pathways, such as enhanced rehabilitation programs, to address these disparities effectively.

At present, there are no standardised definitions for return to normal activity or recovery after surgery. This results in the heterogeneity and limiting interpretation of the data available. In addition, the focus of current evidence have been mainly from high-income countries [14]. These have included return to independence, work, or general mobility. However, definitions vary widely, and there is currently no universally accepted metric for “return to normal activity. Therefore, there is no data to date from low- and middle-income countries. There are selected groups of patients undergoing surgery at higher risk of not returning to normal activity. First, patients with ASA grades III to V were the largest groups at risk of not returning to normal activity. These patients who are likely to have multiple long-term conditions or multimorbidity such as diabetes and hypertension. These patients are likely to have increased risk of complications, both from surgery and underlying multimorbidity [15–17]. Second, patients who undergo contaminated or dirty surgery are at risk of surgical site infections, predisposing to prolonged stay in hospital and need for multiple medical review in their recovery [18, 19]. Finally, patients undergoing midline laparotomies or major or cancer surgery were associated with lower rates of return to normal activities. This may be due to higher rates of complications [20, 21]. Importantly, delayed recovery from surgery may impact on ability to receive chemotherapy, hence best cancer care and lowering risk of cancer-free survival. Collectively, these patient cohorts amplify the need for appropriate pathways and strategies to improve recovery after surgery.

The main strength of this study is the use of a large dataset from a robustly conducted randomised controlled trial. This ensures validity of the data collected, enhancing reliability of the study findings. To the first of our knowledge, this is the first study to evaluate the prevalence of return to normal activities in LMICs, where previously published data have been from high income countries. However, there are important limitations to recognise. First, defining return to normal activity is challenging, since no standardisation exist. Therefore, we have used a self-reporting criterion, which may lead to variation between patients. Second, follow-up was done via a mixture of both in-person and telephone, which may create some variability. However, telephone follow-up constituted for 81.8% (12303/17599) of the data, however we did not pre-plan an analysis by telephone follow up. Although variability in the methods of assessment may influence outcome, telephone method of follow up has been shown to very effective in detecting outcomes such as SSI [22]. Further, follow-up beyond 30-days were not performed. Therefore, long-term outcomes are not available for this cohort, warranting the need in future studies. Finally, there may be potentially unmeasured confounders in the present study which has not been accounted for such as continuous representation of age, baseline functional status and overall complications. Although we have accounted for surgical site infections, overall complications more broadly were not captured in this study and therefore not accounted for. In the context of baseline functional status, the impact of return to normal activities in patients who started off with poor functional status remains unaddressed in the present study.

This study has several implications to both policy and practice. This study highlights the urgent need to strengthen perioperative care and rehabilitation systems within LMICs, particularly in the at groups of lower rates of return to normal activities. Embedding rehabilitation pathways within current perioperative care systems, both in-hospital and out-of-hospital are important towards achieving universal health coverage [14]. At present, adoption of rehabilitation globally is variable and limited [23]. Therefore, inclusion of rehabilitation in the policy development for surgical care, will both improve outcomes for these patients, but also for the wider patients requiring them within health systems. To accelerate progress in the development of surgery-related rehabilitation in low- and middle-income countries, there is a need for greater collaboration between surgery and rehabilitation professionals, and across the health, education and labour sectors. Rehabilitation needs to be integrated into national surgery policies. These can be guided through thee national surgical plan framework, which spans infrastructure, workforce, service delivery, financing and information management [4]. 

To drive change in this area, high-quality research is needed on further areas to improve outcomes for patients. First, longitudinal studies are needed to understand long-term recovery outcomes and the factors influencing these outcomes in LMICs. Such studies can provide deeper insights into the efficacy of different postoperative care interventions and help refine existing protocols. Second, high-quality evidence should focus on evaluating the effectiveness of various rehabilitation interventions in improving recovery outcomes. Evidence from randomised controlled trials assessing different rehabilitation techniques, such as physical therapy, nutritional support, and psychosocial interventions, through different modalities such as e-Health [24] can provide robust evidence to inform clinical practice. Finally, enhancing use of implementation science in research design and conduct may hasten delivery into routine practice rapidly, maximising benefit to patients globally. These include understanding barriers and relevant strategies, improving overall compliance for the end-users.

In summary, this study highlights recovery after abdominal surgery is a problem in low- and middle- income countries, with groups of patients at higher risk. This amplifies the critical need for improving postoperative care in LMICs to improve recovery after surgery. Collaborations across policymakers and healthcare professionals are needed to strengthen rehabilitation programs. By addressing the identified gaps and challenges, it is possible to reduce the societal and economic burdens of delayed recovery, ultimately improving the quality of life for these patients.

## Electronic supplementary material

Below is the link to the electronic supplementary material.

Supplementary Material 1

## Data Availability

The data can be provided on request from the corresponding author.
