# Supplementary material for: Return to normal activity after abdominal surgery: a pre-planned secondary analysis of a randomised controlled trial across seven low- and middle-income countries
Source: BMC Surg. 2025 Aug 29;25:399. doi: 10.1186/s12893-025-03079-1 (PMC12395667; doi:10.1186/s12893-025-03079-1)
Supplement: Supplementary file 1 — Supplementary Material 1 [file 12893_2025_3079_MOESM1_ESM.docx]

**Return to normal activity after abdominal surgery: A pre-planned secondary analysis of a randomised controlled trial across seven low- and middle-income countries**

*NIHR Global Health Research Unit on Global Surgery*

**Collaborating authors are available in the Appendix*

**Corresponding author**

Mr Omar Omar, NIHR Global Health Research Unit on Global Surgery, Institute of Applied Health Research, University of Birmingham, Birmingham, United Kingdom. Email: [O.Omar@bham.ac.uk](mailto:O.Omar@bham.ac.uk)

and

Mr Sivesh Kathir Kamarajah, NIHR Doctoral Fellow, NIHR Global Health Research Unit on

Global Surgery, Institute of Applied Health Research, University of Birmingham,

Birmingham, United Kingdom. Email: [s.k.kamarajah@bham.ac.uk](mailto:s.k.kamarajah@bham.ac.uk)

**Word count:**2,466

**Abstract word count:**314

**Keywords:** abdominal surgery; return to normal activity; return to work

**Short title:** Death after surgery in LMICs

**Conflict of interest:** There are no conflicts of interest to declare.

**Funding:** This research was funded by a National Institute for Health and Care Research (NIHR) Global Health Research Unit Grant (NIHR 16.136.79). The views expressed are those of the authors and not necessarily those of the NIHR or the UK Department of Health and Social Care.

**Data sharing:** Data sharing requests will be considered by the writing group upon written request to the corresponding author.

Table of Contents

[All Patients 3](#_Toc174876054)

[Table S1. Baseline patient-level characteristics for patients with missing outcome data from the CHEETAH randomised controlled trial. 3](#_Toc174876055)

[Table S2. Baseline operative-level characteristics for patients with missing outcome data from the CHEETAH randomised controlled trial. 4](#_Toc174876056)

[Figure S1: Bayesian multivariable regression analysis with multiple imputation for missing data 5](#_Toc174876057)

[Sensitivity analysis by age groups 6](#_Toc174876058)

[Table S3. Subgroup analysis for primary outcome for children (<18 years old) undergoing abdominal surgery in the main CHEETAH randomised controlled trial 6](#_Toc174876059)

[Table S4. Subgroup analysis for primary outcome for adults (>18 years old) undergoing abdominal surgery in the main CHEETAH randomised controlled trial 7](#_Toc174876060)

[Sensitivity analysis by patient gender 8](#_Toc174876061)

[Table S5. Subgroup analysis for primary outcome for male patients only undergoing abdominal surgery in the main CHEETAH randomised controlled trial 8](#_Toc174876062)

[Table S6. Subgroup analysis for primary outcome for female patients only undergoing abdominal surgery in the main CHEETAH randomised controlled trial 9](#_Toc174876063)

[Figure S2. Interaction analysis between planned key factors, adjusted for patient- and operative-level characteristics 10](#_Toc174876064)

[Figure S3. Posterior distribution for model parameters 11](#_Toc174876065)

[Figure S4. Area under the curve 12](#_Toc174876066)

[Figure S5. Trace plot 13](#_Toc174876067)

[Appendix 14](#_Toc174876068)

[List of authors (all groups listed alphabetically by surname) 14](#_Toc174876069)

# All Patients

## Table S1. Baseline patient-level characteristics for patients with missing outcome data from the CHEETAH randomised controlled trial.

|  |  | Total  (n=961) |
| --- | --- | --- |
| Age Group | Adult | 871 (90.6) |
|  | Child | 90 (9.4) |
|  | *Missing* | 0 |
| Sex | Male | 560 (58.3) |
|  | Female | 401 (41.7) |
|  | *Missing* | 0 |
| Timing of surgery | Elective | 263 (27.4) |
|  | Emergency | 698 (72.6) |
|  | *Missing* | 0 |
| Surgery Type | Appendicectomy | 42 (4.4) |
|  | Colorectal | 142 (14.8) |
|  | Gynaecology | 52 (5.4) |
|  | Hepatobiliary | 41 (4.3) |
|  | Laparotomy | 227 (23.6) |
|  | Small bowel | 158 (16.4) |
|  | Upper GI | 139 (14.5) |
|  | Urology | 11 (1.1) |
|  | Other | 149 (15.5) |
|  | *Missing* | 0 |
| Grade of surgery | Minor | 195 (20.3) |
|  | Major | 766 (79.7) |
|  | *Missing* | 0 |
| Diabetes | No | 880 (91.6) |
|  | Yes | 81 (8.4) |
|  | *Missing* | 0 |
| Smoking | Never smoked | 782 (81.4) |
|  | Ex-smoker | 71 (7.4) |
|  | Current smoker | 108 (11.2) |
|  | *Missing* | 0 |
| HIV status | Negative | 486 (50.6) |
|  | Positive | 32 (3.3) |
|  | Not known | 443 (46.1) |
|  | Missing | 0 |

## Table S2. Baseline operative-level characteristics for patients with missing outcome data from the CHEETAH randomised controlled trial.

|  |  | Total  (n=961) |
| --- | --- | --- |
| Indication | Benign | 664 (69.1) |
|  | Malignant | 217 (22.6) |
|  | Trauma | 80 (8.3) |
|  | *Missing* | 0 |
| ASA Grade | Grade I – II | 505 (52.5) |
|  | Grade III-V | 456 (47.5) |
|  | *Missing* | 0 |
| WHO Checklist | No | 39 (4.1) |
|  | Yes | 922 (95.9) |
|  | *Missing* | 0 |
| Pulse Oximetry | No | 3 (0.3) |
|  | Yes | 958 (99.7) |
|  | *Missing* | 0 |
| Prophylactic Antibiotics | No | 16 (1.7) |
|  | Yes | 945 (98.3) |
|  | *Missing* | 0 |
| Surgical Approach | Midline | 849 (88.3) |
|  | Non-Midline | 112 (11.7) |
|  | *Missing* | 0 |
| Contamination | Clean + Clean-contaminated | 352 (36.6) |
|  | Contaminated | 238 (24.8) |
|  | Dirty | 371 (38.6) |
|  | Missing | 0 |
| Change of gloves/instruments | No | 533 (55.5) |
|  | Yes | 428 (44.5) |
|  | *Missing* | 0 |

## Figure S1: Bayesian multivariable regression analysis with multiple imputation for missing data


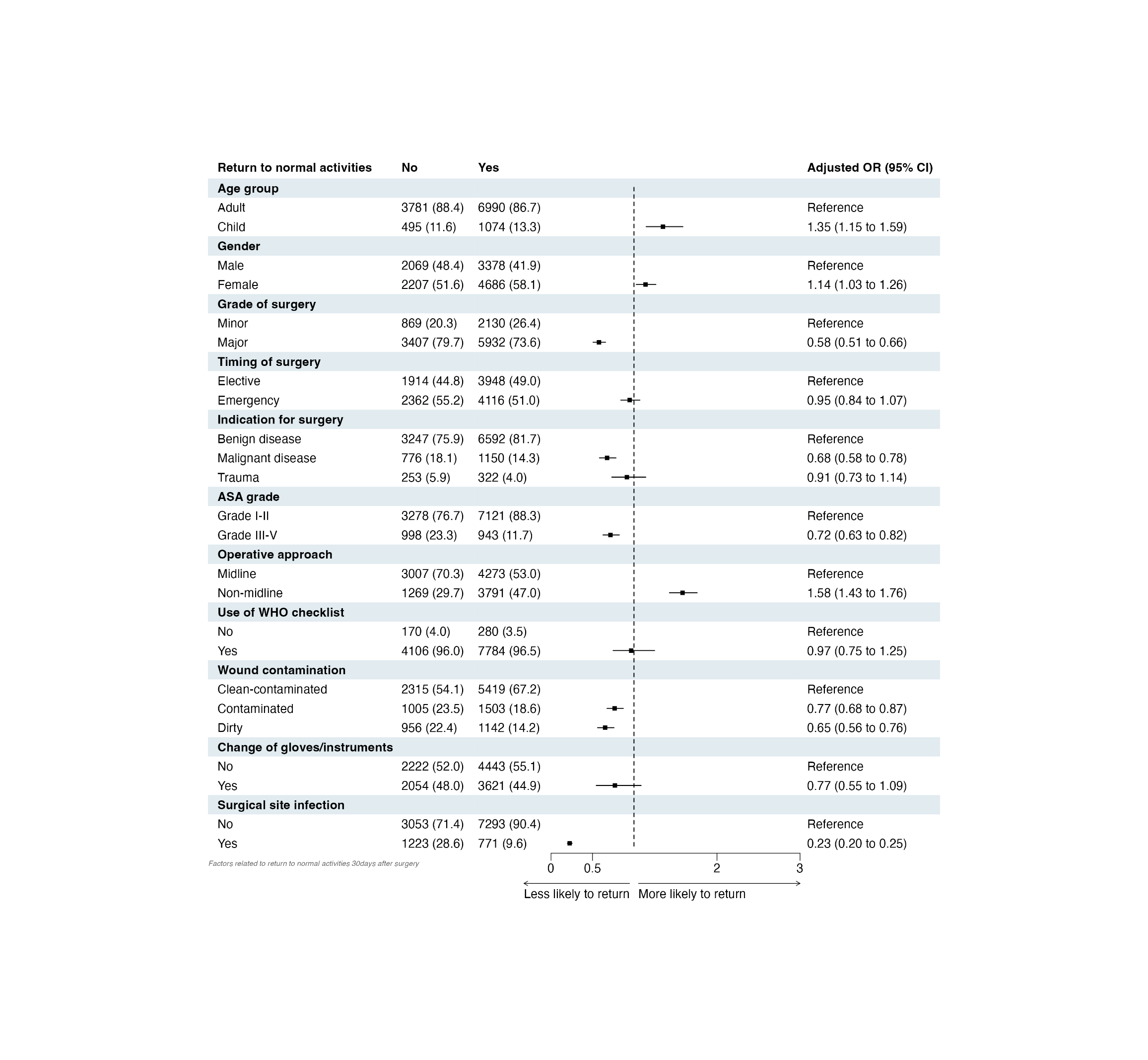


# Sensitivity analysis by age groups

## Table S3. Subgroup analysis for primary outcome for children (<18 years old) undergoing abdominal surgery in the main CHEETAH randomised controlled trial

| **Factor** | **Not fully returned to normal activities**  **(No)** | **Fully returned to normal activities**  **(Yes)** | **Odds ratio (95% credible interval)** |
| --- | --- | --- | --- |
| **Gender** |  |  |  |
| Male | 315 (63.6) | 656 (61.1) | Reference |
| Female | 180 (36.4) | 418 (38.9) | 1.10 (0.83 to 1.47) |
| **Grade of surgery** |  |  |  |
| Minor | 173 (34.9) | 571 (53.2) | Reference |
| Major | 322 (65.1) | 502 (46.8) | 0.51 (0.36 to 0.72) |
| **Timing of surgery** |  |  |  |
| Elective | 110 (22.2) | 244 (22.7) | Reference |
| Emergency | 385 (77.8) | 830 (77.3) | 1.04 (0.70 to 1.56) |
| **Indication for surgery** |  |  |  |
| Benign | 431 (87.1) | 999 (93.0) | Reference |
| Malignant | 17 (3.4) | 27 (2.5) | 0.92 (0.41 to 2.09) |
| Trauma | 47 (9.5) | 48 (4.5) | 0.80 (0.46 to 1.41) |
| **ASA Grade** |  |  |  |
| Grade I – II | 373 (75.4) | 946 (88.1) | Reference |
| Grade III-V | 122 (24.6) | 128 (11.9) | 0.72 (0.48 to 1.09) |
| **Surgical Approach** |  |  |  |
| Midline | 287 (58.0) | 447 (41.6) | Reference |
| Non-Midline | 208 (42.0) | 627 (58.4) | 1.31 (0.95 to 1.81) |
| **Use of WHO Checklist** |  |  |  |
| No | 35 (7.1) | 78 (7.3) | Reference |
| Yes | 460 (92.9) | 996 (92.7) | 0.84 (0.44 to 1.52) |
| **Wound contamination** |  |  |  |
| Clean-contaminated | 175 (35.4) | 556 (51.8) | Reference |
| Contaminated | 127 (25.7) | 255 (23.7) | 0.65 (0.44 to 0.95) |
| Dirty | 193 (39.0) | 263 (24.5) | 0.49 (0.33 to 0.73) |
| **Change of gloves/instrument** |  |  |  |
| No | 208 (42.0) | 529 (49.3) | Reference |
| Yes | 287 (58.0) | 545 (50.7) | 0.78 (0.43 to 1.42) |
| **SSI** |  |  |  |
| No | 340 (68.7) | 963 (89.7) | Reference |
| Yes | 155 (31.3) | 111 (10.3) | 0.21 (0.14 to 0.30) |

## Table S4. Subgroup analysis for primary outcome for adults (>18 years old) undergoing abdominal surgery in the main CHEETAH randomised controlled trial

| **Factor** | **Not fully returned to normal activities**  **(No)** | **Fully returned to normal activities**  **(Yes)** | **Odds ratio (95% credible interval)** |
| --- | --- | --- | --- |
| **Gender** |  |  |  |
| Male | 1754 (46.4) | 2722 (38.9) | Reference |
| Female | 2027 (53.6) | 4268 (61.1) | 1.15 (1.03 to 1.29) |
| **Grade of surgery** |  |  |  |
| Minor | 696 (18.4) | 1559 (22.3) | Reference |
| Major | 3085 (81.6) | 5430 (77.7) | 0.57 (0.49 to 0.65) |
| **Timing of surgery** |  |  |  |
| Elective | 1804 (47.7) | 3704 (53.0) | Reference |
| Emergency | 1977 (52.3) | 3286 (47.0) | 0.96 (0.84 to 1.09) |
| **Indication for surgery** |  |  |  |
| Benign | 2816 (74.5) | 5593 (80.0) | Reference |
| Malignant | 759 (20.1) | 1123 (16.1) | 0.61 (0.52 to 0.72) |
| Trauma | 206 (5.4) | 274 (3.9) | 0.93 (0.72 to 1.19) |
| **ASA Grade** |  |  |  |
| Grade I – II | 2905 (76.8) | 6175 (88.3) | Reference |
| Grade III-V | 876 (23.2) | 815 (11.7) | 0.73 (0.63 to 0.84) |
| **Surgical Approach** |  |  |  |
| Midline | 2720 (71.9) | 3826 (54.7) | Reference |
| Non-Midline | 1061 (28.1) | 3164 (45.3) | 1.59 (1.42 to 1.78) |
| **Use of WHO Checklist** |  |  |  |
| No | 135 (3.6) | 202 (2.9) | Reference |
| Yes | 3646 (96.4) | 6788 (97.1) | 0.99 (0.73 to 1.35) |
| **Wound contamination** |  |  |  |
| Clean-contaminated | 2140 (56.6) | 4863 (69.6) | Reference |
| Contaminated | 878 (23.2) | 1248 (17.9) | 0.77 (0.67 to 0.90) |
| Dirty | 763 (20.2) | 879 (12.6) | 0.66 (0.55 to 0.78) |
| **Change of gloves/instrument** |  |  |  |
| No | 2014 (53.3) | 3914 (56.0) | Reference |
| Yes | 1767 (46.7) | 3076 (44.0) | 0.81 (0.54 to 1.19) |
| **SSI** |  |  |  |
| No | 2713 (71.8) | 6330 (90.6) | Reference |
| Yes | 1068 (28.2) | 660 (9.4) | 0.20 (0.17 to 0.23) |

# Sensitivity analysis by patient gender

## Table S5. Subgroup analysis for primary outcome for male patients only undergoing abdominal surgery in the main CHEETAH randomised controlled trial

| **Factor** | **Not fully returned to normal activities**  **(No)** | **Fully returned to normal activities**  **(Yes)** | **Odds ratio (95% credible interval)** |
| --- | --- | --- | --- |
| **Age group** |  |  |  |
| Adult | 1754 (84.8) | 2722 (80.6) | Reference |
| Child | 315 (15.2) | 656 (19.4) | 1.39 (1.13 to 1.71) |
| **Grade of surgery** |  |  |  |
| Minor | 512 (24.7) | 1237 (36.6) | Reference |
| Major | 1557 (75.3) | 2139 (63.4) | 0.51 (0.43 to 0.61) |
| **Timing of surgery** |  |  |  |
| Elective | 702 (33.9) | 1249 (37.0) | Reference |
| Emergency | 1367 (66.1) | 2129 (63.0) | 0.94 (0.77 to 1.13) |
| **Indication for surgery** |  |  |  |
| Benign | 1527 (73.8) | 2635 (78.0) | Reference |
| Malignant | 365 (17.6) | 497 (14.7) | 0.56 (0.44 to 0.70) |
| Trauma | 177 (8.6) | 246 (7.3) | 0.98 (0.74 to 1.30) |
| **ASA Grade** |  |  |  |
| Grade I – II | 1552 (75.0) | 2889 (85.5) | Reference |
| Grade III-V | 517 (25.0) | 489 (14.5) | 0.68 (0.56 to 0.82) |
| **Surgical Approach** |  |  |  |
| Midline | 1549 (74.9) | 2024 (59.9) | Reference |
| Non-Midline | 520 (25.1) | 1354 (40.1) | 1.47 (1.24 to 1.76) |
| **Use of WHO Checklist** |  |  |  |
| No | 106 (5.1) | 135 (4.0) | Reference |
| Yes | 1963 (94.9) | 3243 (96.0) | 1.35 (0.94 to 1.94) |
| **Wound contamination** |  |  |  |
| Clean-contaminated | 868 (42.0) | 1793 (53.1) | Reference |
| Contaminated | 589 (28.5) | 872 (25.8) | 0.80 (0.67 to 0.96) |
| Dirty | 612 (29.6) | 713 (21.1) | 0.65 (0.53 to 0.80) |
| **Change of gloves/instrument** |  |  |  |
| No | 1034 (50.0) | 1812 (53.6) | Reference |
| Yes | 1035 (50.0) | 1566 (46.4) | 0.91 (0.58 to 1.43) |
| **SSI** |  |  |  |
| No | 1423 (68.8) | 2977 (88.1) | Reference |
| Yes | 646 (31.2) | 401 (11.9) | 0.22 (0.18 to 0.26) |

## Table S6. Subgroup analysis for primary outcome for female patients only undergoing abdominal surgery in the main CHEETAH randomised controlled trial

| **Factor** | **Not fully returned to normal activities**  **(No)** | **Fully returned to normal activities**  **(Yes)** | **Odds ratio (95% credible interval)** |
| --- | --- | --- | --- |
| **Age group** |  |  |  |
| Adult | 2027 (91.8) | 4268 (91.1) | Reference |
| Child | 180 (8.2) | 418 (8.9) | 1.47 (1.13 to 1.91) |
| **Grade of surgery** |  |  |  |
| Minor | 357 (16.2) | 893 (19.1) | Reference |
| Major | 1850 (83.8) | 3793 (80.9) | 0.58 (0.47 to 0.71) |
| **Timing of surgery** |  |  |  |
| Elective | 1212 (54.9) | 2699 (57.6) | Reference |
| Emergency | 995 (45.1) | 1987 (42.4) | 0.95 (0.79 to 1.13) |
| **Indication for surgery** |  |  |  |
| Benign | 1720 (77.9) | 3957 (84.4) | Reference |
| Malignant | 411 (18.6) | 653 (13.9) | 0.68 (0.55 to 0.84) |
| Trauma | 76 (3.4) | 76 (1.6) | 0.78 (0.51 to 1.19) |
| **ASA Grade** |  |  |  |
| Grade I – II | 1726 (78.2) | 4232 (90.3) | Reference |
| Grade III-V | 481 (21.8) | 454 (9.7) | 0.79 (0.65 to 0.96) |
| **Surgical Approach** |  |  |  |
| Midline | 1458 (66.1) | 2249 (48.0) | Reference |
| Non-Midline | 749 (33.9) | 2437 (52.0) | 1.61 (1.40 to 1.87) |
| **Use of WHO Checklist** |  |  |  |
| No | 64 (2.9) | 145 (3.1) | Reference |
| Yes | 2143 (97.1) | 4541 (96.9) | 0.66 (0.45 to 0.97) |
| **Wound contamination** |  |  |  |
| Clean-contaminated | 1447 (65.6) | 3626 (77.4) | Reference |
| Contaminated | 416 (18.8) | 631 (13.5) | 0.73 (0.60 to 0.87) |
| Dirty | 344 (15.6) | 429 (9.2) | 0.61 (0.48 to 0.77) |
| **Change of gloves/instrument** |  |  |  |
| No | 1188 (53.8) | 2631 (56.1) | Reference |
| Yes | 1019 (46.2) | 2055 (43.9) | 0.73 (0.45 to 1.19) |
| **SSI** |  |  |  |
| No | 1630 (73.9) | 4316 (92.1) | Reference |
| Yes | 577 (26.1) | 370 (7.9) | 0.17 (0.14 to 0.20) |

##
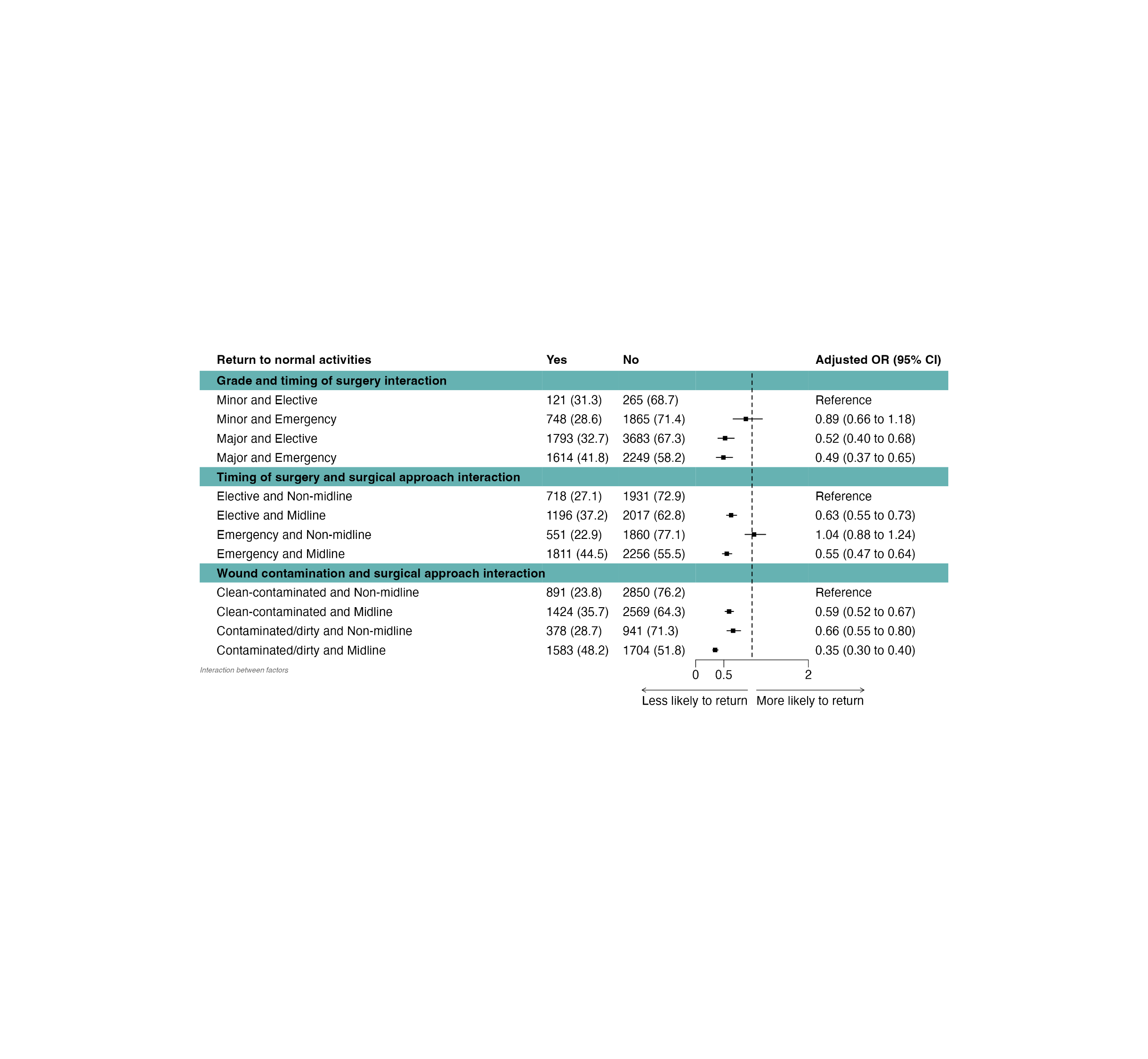
Figure S2. Interaction analysis between planned key factors, adjusted for patient- and operative-level characteristics

## Figure S3. Posterior distribution for model parameters

**
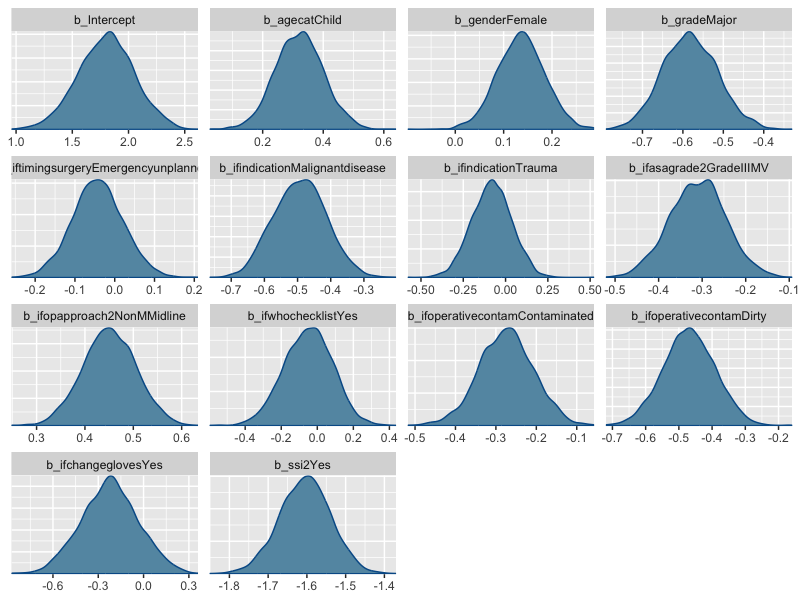
**

## Figure S4. Area under the curve

**
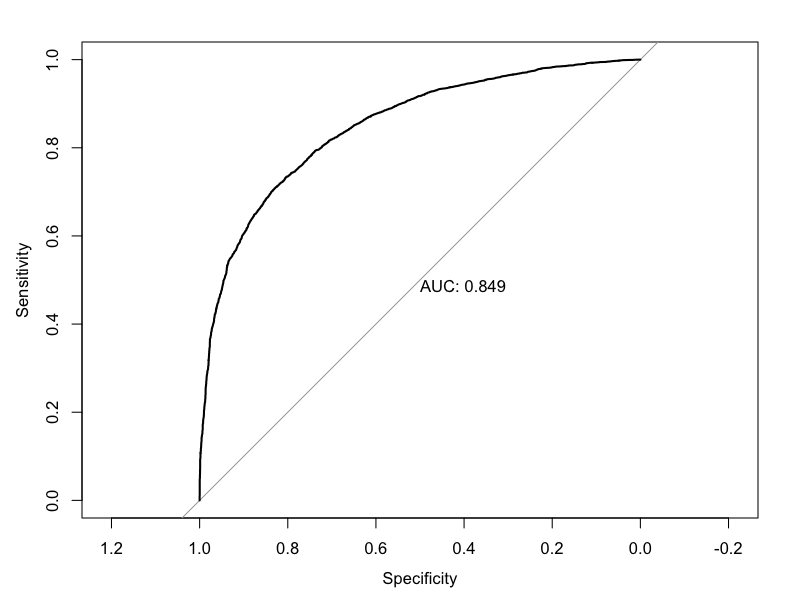
**

## Figure S5. Trace plot

**
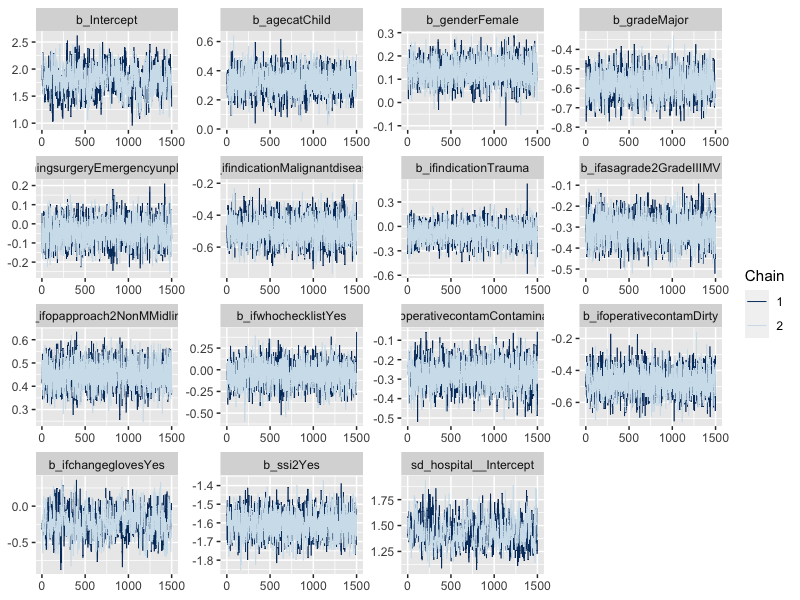
**

**Trace plot is a common check for the Markov Chain Monte Carlo sampler convergence. The plots above show the behaviour of the simulations (i.e., the chains) used to approximate the posterior distribution, where the x-axis represents the number of iterations and the y-axis the value of the parameter. To ensure the reliability of a finite set of samples, we routinely run several chains, i.e. we start the procedure at different random initial starting points and check whether the different chains have converged to stable values. In the figure above, the chains appear to be indistinguishable except for random noise and have converged to stable values. If the chains had not converged, the trajectories of the chains would be in different directions.*

# Appendix A. Return to normal activity

# Since your surgery, have you returned to your normal activities? (Yes / No)

# Do you feel your ability to perform daily tasks is the same as before your surgery? (Yes / No)

# Are you currently working / attending school / managing your usual responsibilities? (Yes / No)

# Appendix B. Authorship list

**Writing group:** Sivesh K Kamarajah*, Omar Omar*, Adesoji O Ademuyiwa, Dhruva Ghosh, Lawani Ismail, Virginia Ledda, Elizabeth Li, Faustin Ntirenganya, Stephen Tabiri, Aneel Bhangu

*both authors contributed equally

**Trial Management Group:** Adesoji O Ademuyiwa, Aneel Bhangu, Peter Brocklehurst, Sohini Chakrabortee, Dhruva Ghosh, James Glasbey, Pollyanna Hardy, Ewen Harrison, Lawani Ismail, Bryar Kadir, Rachel Lillywhite, Laura Magill, Antonio Ramos de la Medina, Rachel Moore, Mark Monahan, Dion Morton, Dmitri Nepogodiev, Faustin Ntirenganya, Omar Omar, Thomas Pinkney, Donna Smith, Stephen Tabiri, Neil Winkles

**Statistical analysis:** Omar Omar

**Data handling and governance:** Aneel Bhangu, Felicity Brant, Elizabeth Li, Rachel Lillywhite, Maria Picciochi, Donna Smith

**Collaborators** (*denotes the hospital Principle Investigator):

**Benin**

Centre Hosptalier Departemental de l’Atacora: Didier Ahogni, Aristide Ahounou, K. Alassan Boukari, Oswald Gbehade, Thierry K Hessou, Sinama Nindopa, M.J. Bienvenue Nontonwanou, Nafissatou Orou Guessou, Arouna Sambo, Sorekou Victoire Tchati, Affisatou Tchogo, Semevo Romaric Tobome*, Parfait Yanto

Centre Hospitalier Universitaire et Departemental du Borgou et Alibori: Isidore Gandaho, Armel Hadonou, Simplice Hinvo, Montcho Adrien Hodonou*, Sambo Bio Tamou

Centre Hospitalier Universitaire et Departemental Oueme-Plateau: Souliath Lawani, Covalic Melic Bokossa Kandokponou, Francis Moise Dossou, Antoine Gaou, Roland Goudou, Marie-Claire Kouroumta, Ismail Lawani*, Enrif Malade, Anne stredy Mkoh Dikao, Joel Nzuwa Nsilu, Pencome Ogouyemi

Centre Hospitalier Universitaire de Zone de Suru Lere: Marcelin Akpla, Nathan Bisimwa Mitima, Blaise Kovohouande, Cyrille Kpangon*, Stephane Laurent Loupeda

Hopital Bethesda: Mamonde Victorin Agbangla, Sena Emmanuel Hedefoun, Thierry Mavoha*, Juvenal Ngaguene, Janvier Rugendabanga, Rish Romaric Soton, Martin Totin

Hopital de Zone de Dassa-Zoume: Mouhamed Agbadebo*, Irene Akpo, Hubert Dewamon, Martin Djeto, Aissatou Hada, Monsede Hollo, Albert Houndji, Anasthasie Houndote, Sylvestre Hounsa, Expedit Kpatchassou, Hugues Yome,

Hopital de Zone de Kandi: Mohamed Moussa Alidou, Eric Jerry Bara*, B.T. Bonheur Dossou Yovo, Robert Guinnou, Souleymane Hamadou, H.Pauline Kola, Nabil Moussa

Hopital de Zone de Klouekanme: Boniface Cakpo, Lolyta Etchisse, Emery Hatangimana, Moise Muhindo, Katia Sanni, Agossou Barthelemy Yevide*

Hopital de Zone de Menontin: Hermann Agossou, Fiston Basirwa Musengo, Hulrich Behanzin*, Djifid Morel Seto

Hopital de Zone de OUIDAH: Bill Armstrong Alia*, Arnaud Alitonou, Y.Edith Mehounou

Hopital d’Instruction des Armees de Parakou: Lucien Agbanda, Julien Attinon, Marcel Gbassi, Nounagnon Rene Hounsou*

Ghana

Berekum Holy Family Hospital: Regina Acquah, Charles Banka, Derick Esssien, Romeo Hussey*, Yakubu Mustapha, Kojo Nunoo-Ghartey, Grace Yeboah

Cape Coast Teaching Hospital: Luke A Aniakwo, Margarey N M Adjei, Yvonne Adofo-Asamoah, Meshach M Agyapong, Thomas Agyen, Baba A B Alhassan, Mabel P Amoako-Boateng, Anthony Baffour Appiah, Josephine Ashong, Joseph K Awindaogo, Benjamin B Brimpong, Makafui S C J K Dayie, Donald Enti, Wendy W Ghansah, Jude E Gyamfi, Patience Koggoh, Richard Kpankpari, Vincent Kudoh, Samuel Mensah, Philip Mensah, Isabella N Morkor Opandoh, Martin T Morna*, Michael Nortey, Emelia Odame, Emmanuel O Ofori, Sandra Quaicoo, Elizabert M Quartson, Cynthia Teye-Topey, Makafui Yigah, Safia Yussif

Eastern Regional Hospital: Esther Adjei-Acquah, Vera O Agyekum-Gyimah, Eric Agyemang, Arko Akoto- Ampaw, Forster Amponsah-Manu*, Temitope E Arkorful, Moses A Dokurugu, Nanabanyin Essel, Aja Ijeoma, Emmanuel L Obiri, Richard Ofosu-Akromah, Karen N D Quarchey

Effia-Nkwanta Regional Hospital: Leslie Adam-Zakariah*, Aaron B Andoh, Esther Asabre, Ruby A Boateng, Barbara Koomson, Atta Kusiwaa, Adeline Naah, Ato Oppon-Acquah, Benjamin A Oppong

Greater Accra Regional Hospital: Emma A Agbowada, Ameley Akosua, Ralph Armah*, Christopher Asare, Lawrence K B Awere-Kyere, Amanda Bruce-Adjei, Nana Ama Christian, Delali A Gakpetor, Korankye K Kennedy, Jacqueline Mends-Odro, Ambe Obbeng, Doris Ofosuhene, Dorcas Osei-Poku, Zelda Robertson

Komfo Anokye Teaching Hospital: Dorcas O Acheampong, Jane Acquaye, Juliana Appiah, Joshua Arthur, Jonathan Boakye-Yiadom, Anita Eseenam Agbeko, Frank E Gyamfi*, Bertina B Nyadu

Korle Bu Teaching Hospital: Samira Abdulai, Nii A Adu-Aryee, Nelson Agboadoh, Erica Akoto, Joachim K Amoako, Nicholas T Aperkor, Wilfred K Asman, Godsway S Attepor, Antoinette A Bediako-Bowan*, Kwaku Boakye-Yiadom, George D Brown, Florence Dedey, Victor K Etwire, Benjamin S Fenu, Philemon K Kumassah, Linda A Larbi-Siaw, Josephine Nsaful, David O Olatola, Sandra E Tsatsu, Theodore Wordui

Salaga Municipal Hospital: Iddrisu I A Abdul-Aziz, Fatao Abubakari, Johnson Akunyam, Gilbert A G Anasara, Cletus Ballu, Charles G Barimah, Guy C Boateng, Ponala W Kwabena, Seidu M Kwarteng, Prosper T Luri*, Kennedy Ngaaso, David K D Ogudi

Sunyani Regional Hospital: Vivian Adobea, Amos Bennin, Stanley Doe, Ruth Sarfo Kantanka, Ephraim Kobby, Collins Kyeremeh, Edwin Osei, Prince Yeboah Owusu, Frank Owusu*, Clement Sie-Broni, Marshall Zume

Tamale Teaching Hospital (Hub): Saba Abdul-Hafiz, Daniel K Acquah, Shamsudeen M Adams, Mohammed S Alhassan, Munira Amadu, Samuel A Asirifi, Martin Awe, Millicent Azanlerigu, Mathias K Dery, Yenli Edwin, Abantanga Atindaana Francis, Gbana Limann, Aloysius Maalekuu, Hawa Malechi, Sheriff Mohammed*, Ibrahim Mohammed, Kareem Mumuni, Bernard A Ofori, Jonathan I K Quansah, Napoleon Bellua Sam, Anwar S Seidu, Stephen Tabiri*, Shekira Yahaya

Techiman Holy Family Hospital: Emmanuel Kojo Acquah, Jaabir Alhassan, Percy Boakye, Christian L Coompson*, Addo K Gyambibi, Ametepe Jeffery-Felix, Bismark E Kontor, Ruth Manu, Elijah Mensah, Gifty Naah, Carmen Noufuentes, Abraham Sakyi

India

All India Institute of Medical Science, Jodhpur: Ramkaran Chaudhary, Sanjeev Misra*, Puneet Pareek, Manish Pathak, Dharma R Poonia, Kirti K Rathod, Mahaveer S Rodha, Naveen Sharma, Nivedita Sharma, Subhash C Soni, Vaibhav K Varsheney, Jeevan R Vishnoi

All India Institute of Medical Science, Rishikesh: Deepak K Garnaik, Farhanul Huda, Manoj J Lokavarapu, Neha Mishra, Rohit Ranjan, Rajkumar K Seenivasagam*, Shanky Singh, Pratik Solanki, Raunak Verma, Enono Yhoshu

Baptist Hospital Tezpur: Suzan John, Jeffery A Kalyanapu, Ananta Kutma, Sanish Philips*

Christian Hospital Chinchpada: Arun K Gautham, Alice Hepzibah, Grace Mary, Deepak S Singh*

Christian Hospital Madhipura: Eunice S Abraham, Chetana Chetana, Amos Dasari, Prashant Dummala, Chinta S Gold, Jurgen Jacob, Jeremiah N Joseph, Elizabeth N Kurien, Priya Mary, Arpit J Mathew*, Amy E Mathew, Danita D Prakash, Oliver Samuel, Ashwin Sukumar, Niyah Syam, Rose Varghese

Christian Medical College & Hospital, Ludhiana (Hub): Alisha Bhatt, William Bhatti, Tapasya Dhar, Dhruva N Ghosh, Ankush Goyal, Sunita Goyal, Monika A Hans, Parvez D Haque, Deepak Jain, Rita Jain, Jyoti Jyoti, Savleen Kaur, Karan Kumar, Anil Luther*, Amit Mahajan, Kavita Mandrelle, Vishal Michael, Partho Mukherjee, Reuben Rajappa, Vivin Daniel Sam, Prashant Singh, Atul Suroy, Ravinder Singh Thind, Sreejith K Veetil, Rahul Williams

Christian Medical College & Hospital, Vellore: Sreekar D, Esther R Daniel, Smitha E Jacob, Mark R Jesudason, Pushplatha Kumari, Rohin Mittal*, Soosan Prasad, Vasanth Mark Samuel, Bharat Shankar, Srujan Sharma, Moonish V Sivakumar, Suraj Surendran, Anita Thomas, Paul Trinity

GB Pant Institute of Postgraduate Medical Education and Research: Sudheer Kanchodu, K Leshiini, Sundeep S Saluja*

GMC Chandigargh: Ashok K Attri*, Ishan Bansal, Sanjay Gupta, Monika Gureh, Simran Kapoor

GMC Patiala: Manisha Aggarwal, Vinoth Kanna, Harmanjot Kaur, Ashwani Kumar*, Simrandeep Singh, Gurtaj Singh

Herbertpur Christian Hospital: Viju John*

Kalpana Chawla GMC: Mohammed Adnan, Nivesh Agrawal, Uttkarsh Kumar, Pardeep Kumar, Abhishek S, Vikram Sehrawat, Deepak Singla, Gaurav Thami*

Kasturba Medcial College Hospital, Manipal: Vijay Kumar*, Stanley Mathew, Murlidhar V Pai, P S Prabhu, Sundeep P T

King George’s Medical Unit: Naseem Akhtar, Arun Chaturvedi, Sameer Gupta, Vijay Kumar, Puneet Prakash, Shiv Rajan*, Mohit Singh, Abhilasha Tripathi

Lady Willingdon Hospital, Manali: Philip V Alexander*, Josy Thomas, Pradeep Zechariah Makunda Christian Leprosy & General Hospital: Vijay A Ismavel, Moloti Kichu*, Carolin V Solomi Padhar Hospital: Rahul A Alpheus, Ashish Victor Choudhrie*, Rashmi Jacob Gunny, Susan Joseph PGI Chandigargh: Muneer A Malik, Nitin J Peters*, Neha Pundir, Ram Samujh

Sher-I Kashmir Institute of Medical Sciences: Hafsa I Ahmed, Gowhar Aziz, Nisar A Chowdri, Rayees A Dar, Robindera Kour, Imtiyaz Mantoo, Asif Mehraj*, Fazl Q Parray, Najmus Saqib, Zamir A Shah, Rauf A Wani

St Stephens Hospital: Subrat Raul*, Komal Rautela, Rajeev Sharma, Nishu Singh, Rakesh Vakil

Tata Medical College, Kolkata: Priyanka Chowdhury, Sona Chowdhury, Sonia Mathai, Pragyanmai Nayak, Bipradas Roy*

Mexico

Centro Medico Nacional de Occidente: Andrea S Alvarez Villaseñor, Kriscia V Ascencio Díaz, Victor J Avalos Herrera, Francisco J Barbosa Camacho, Aldo Bernal Hernández, Elyoenai Bonilla Ahumada, Irma V Brancaccio Pérez, Miguel A Calderón Llamas, Guadalupe Castillo Cardiel, Guillermo A Cervantes Cardona, Gabino Cervantes Guevara, Enrique Cervantes Perez, Maria Chávez, Jonathan M Chejfec Ciociano, Luis R Cifuentes Andrade, Ana O Cortés Flores, Edgar J Cortes Torres, Tania A Cueto Valadez, Andrea E Cueto Valadez, Esteban Cueva Martinez, Paulina Domínguez Barradas, Isaac Esparza Estrada, Paola Flores Becerril, Jose A Flores Cardoza, Clotilde Fuentes Orozco, Luis A García González, Benjamín García Reyna, Eduardo Gómez Sánchez, Jaime L González Bojorquez, Eduardo González Espinoza, Alejandro González Ojeda*, Fanny Y González Ponce, Cristhian S Guerrero Ramírez, José A Guzmán Barba, Bertha G Guzmán Ramírez, Mario J Guzmán Ruvalcaba, Daniel A Hérnandez Alva, Silvia A Ibarra Camargo, Juan C Ibarrola Peña, Martin Islas Torres, Jorge Jiménez Tornero, Zayra M Lara Pérez, Roberto Mares País, Mel P Mellado Tellez, Roberto C Miranda Ackerman, Damián Mora Santana, Gilberto Morgan Villela, Rodrigo Nájar Hinojosa, Cesar Nuño Escobar, Itzel Ochoa Rodríguez, Oscar Olvera Flores, Angelica Ortega Barreiro, Jacqueline Osuna Rubio, Luis R Pacheco Vallejo, Víctor H Pérez Bocanegra, Jose V Pérez Navarro, Francisco J Plascencia Posada, María A Quirarte Hernández, Luis R Ramirez Gonzalez, Emilio A Reyes Elizalde, Evelia V Romo Ascencio, Cornelio Ruelas Bravo, Carlos B Ruiz Velasco, José A Sánchez Martínez, Guillermo Sanchez Villaseñor, José I Sandoval Pulido, Alejandro G Serrano García, Luis O Suárez Carreón, Juan J Tijerina Ávila, Jesus O Vega Gastelum, Melissa L Vicencio Ramirez, Maria F Zarate Casas, Carlos J Zuloaga Fernández del Valle

Hospital Civil de Guadalajara: Jesus Antonion Aguilar Mata, Miguel Antonio Calderon Vanegas, Rocio Guadalupe Cano Arias, Carlos Colunga Tinajero, Fernanda Diaz Samano, Fernando Duque Zepeda, Brenda Vanessa Enriquez Barajas, Gerardo Gallardo Banuelos, Marijose De Cristo Gonzalez Calvillo, Francisco Ibanez Ortiz, Maryzela Lazo Ramirez, Gerardo Lopez Arroyo, Laura Olivia Montano Angeles, David Giovanny I Morales Iriarte, Angelo Fernando Mortola Lomeli, Jose Esteban Orozco Navarro, Jaime Orozco Perez, Damaris Orozco Ramirez, Laura Gabriela Pena Baolboa, Jesus Pizarro Lozano, Guillermo Yanowsky Reyes*

Hospital De Especialidades Del Nino Y La Mujer: Monica N Castillo*, Ana Camille G Dominguez, Dorihela H Mellado, Jesus Flavio M Morales, Luz del Carmen M Namur, Jose Alberto A Pesquera

Hospital Espanol Veracruz (Hub): Laura Martinez Perez Maldonado, Antonio Ramos De la Medina

Hospital General Dr. Manuel Gea Gonzalez: Katya Bozada-Gutierrez, Ana Florencia Casado-Zarate, Roberto Delano-Alonso, Jose Herrera-Esquivel, Mucio Moreno-Portillo, Mario Trejo-Avila*

Hospital Regional de Alta Especialidad del Bajio: Roland Kevin Cethorth Fonseca, Edgard Efren Lozada Hernandez*, Bruno Crocco Quiros, Jairo Arturo Rodriguez Ramirez

UMAE Hospital de Pediatria CMNO: Gabriela Ambriz-González*, Mitzi R Becerra Moscoso, Ishtar Cabrera-Lozano, Ana B Calderón-Alvarado, Francisco J León-Frutos, Erick E Villanueva-Martínez

Nigeria

Ahmadu Bello University Teaching Hospital: Aisha Abdullahi, Maimuna Abubakar, Mohammed S Aliyu, Mudi Awaisu, Fadimatu Bakari, Abigail Olajumoke Balogun, Mohammed Bashir, Ahmad Bello, Muhammad Daniyan, Kehinde Michael Duromola, Stephen G Gana, Mukoro Duke George, Justina Gimba, Isaac Gundu, Lambert Onahi Iji, Aminat O Jimoh, Afolabi K Koledade, Ahmad T Lawal, Bilkisu K Lawal, Aisha

Mustapha, Stanley Emeka Nwabuoku, Oluseyi O Ogunsua, Ifeanyi Fidelis Okafor, Ethos Ike Okorie, Nasir Oyelowo, Ibrahim A Saidu, Tunde T Sholadoye, Ibrahim Sufyan, Musliu Adetola Tolani*, Aliyu Muhammad Tukur, Ahmad Shehu Umar, Aminatu M Umar, Hajara Umaru-Sule, Mohammed Usman, Anisah Yahya, Alfa Yakubu, Salisu Abeku Yusuf

Aminu Kano Teaching Hospital: Abdulhafiz A Abdulkarim, Lawal Barau Abdullahi, Muzzammil Abdullahi, Khadija A Ado, Nura U Aliyu, Lofty-John Chukwuemeka Anyanwu*, Sulaiman M Daneji, Mahmoud Kawu Magashi, Mohammad A Mohammad, Abubakar Bala Muhammad, Saminu S Muhammad, Bello Abodunde Muideen, Calistus U Nwachukwu, Suleiman B Sallau, Abdulrahman A Sheshe, Abdulmajeed Soladoye, Idris Usman Takai, Garzali I Umar, Abubakar Yahaya

Barau Dikko Teaching Hospital: Lubabatu Abdulrasheed, Joel A Adze, Lydia R Airede, Bashiru Aminu, Stephen B Bature, Firdaws Bello-Tukur, Damai Chinyio, Sharon A N Duniya, Moses C Galadima, Babatunde K Hamza, Samaila Joshua, Stephen A Kache*, Williams Y Kagomi, Ifeanyi A Kene, Jamila Lawal, Jerry G Makama, Caleb Mohammed, Amina A Mohammed-Durosinlorun, Deborah Nuwam, Danjuma Sale, Abdulrasheed Sani, Salome Tabara, Mathew C Taingson, Emmanuel Usam, Josiah Yakubu

Federal Medical Centre, Owo: Folasade Adegoke, Oluwasuyi Ige, Tunde A Odunafolabi, Chukwuma E Okereke*, Oluwafemi O Oladele, Oluwaseun H Olaleye, Oyetunde O Olubayo

Federal Teaching Hospital, Ido-Ekiti: Olukayode P Abiola, Henry O Abiyere, Idowu O Adebara, Gbadebo T C Adeleye, Adebayo A Adeniyi, Olumide E Adewara, Olabisi T Adeyemo, Ademola A Adeyeye, Abimbola L Ariyibi, Babatunde S Awoyinka, Olumide M Ayankunle, Olakunle F Babalola, Adewumi Bakare, Tajudeen I B Bakare, Oluseyi O Banjo, Peter A Egharevba, Oluwafemi S Fatudimu, John A Obateru, Oluremi J Odesanya, Owolabi D Ojo, Abiodun I Okunlola*, Cecilia K Okunlola, Adewale T Olajide, Tesleem O Orewole, Adedayo I Salawu

Lagos State University Teaching Hospital: Moruf A Abdulsalam, Aderinsola T Adelaja, Olalekan T Ajai, Olukemi Akande, Noble Anyanwu, Kazeem M Atobatele, Oludayo Oluwaseyi Bakare, Grace Eke, Omolara M Faboya, Zainab O Imam, Francisca C Nwaenyi, Ayokunle A Ogunyemi, Mobolaji A Oludara, Olufunmilade A Omisanjo, Chinonso U Onyeka, Olabode A Oshodi, Yusuf A Oshodi, Yemisi Oyewole, Omotade S Salami, Omolara M Williams*

Lagos University Teaching Hospital: Esther Abunimye, Adesoji O Ademuyiwa*, Adebunmi Adeoluwa, Adedotun Adesiyakan, Victoria Ibukunoluwa Adeyeye, Moses Vincent Agbulu, Opeyemi Rebecca Akinajo, David O Akinboyewa, Felix M Alakaloko, Iyabo O Alasi, Michael Amao, Christiana Ashley-Osuzoka, Oluwole A Atoyebi, Olanrewaju S Balogun, Christopher O Bode, Maryam Oluwatobi Busari, Nnamdi Jonathan Duru, Glory Bassey Edet, Olumide A Elebute, Francis Chinonso Ezenwankwo, Adedeji L Fatuga, Christianah Gbenga-Oke, George C Ihediwa, Emmanuel Sylvester Inyang, Adesola I Jimoh, Jubril Oladayo Kuku, Oluwaseun A Ladipo-Ajayi, Abdulrazzaq O Lawal, Ayomide Makanjuola, Christian Chigoze Makwe, Chinelo Victoria Mgbemena, Samuel U Nwokocha, Moses Adebisi Ogunjimi, Ephraim Okwudiri Ohazurike, Rufus W Ojewola, Moyosoluwa Eunice Badedale, Chike J Okeke, Adeyemi A Okunowo, Abraham T Oladimeji, Thomas O Olajide, Olabisi Olanrewaju, Olawunmi Olayioye, Oluwaseun O Oluseye, Stephen Olutola, Kenneth Onyekachi, Adeola Ayoola Orowale, Emili Osariemen, Adedapo Olumide Osinowo, Benedetto Osunwusi, Emmanuel Owie, Christianah Bidemi Oyegbola, Justina O Seyi-Olajide, Adaiah P Soibi-Harry, Manuella Talla Timo, Aloy Okechukwu Ugwu, Emmanuel Ojo Williams

Nnamdi Azikiwe University Teaching Hospital: Innocent O Duruewuru, Ochonma A Egwuonwu, Okechukwu Hyginus Ekwunife*, James J Emeka, Victor Ifeanyichukwu Modekwe, Chimdiebele Daisy Nwosu, Sylvester O Obiechina, Ahuizechukwu E Obiesie, Celestine I Okafor, Theophilus O Okonoboh, Chukwuemeka Okoro, Odili A Okoye, Onyekachi A Onu, Chukwudubem C Onyejiaka, Chisom Faith Uche, Joseph O Ugboajah, Jideofor Okechukwu Ugwu, Kenneth Ugwuanyi, Chuka Ugwunne

Obafemi Awolowo University Teaching Hospitals Complex, Ile-Ife: Akeem A Adeleke, Akinfolarin C Adepiti, Adewale A Aderounmu, Abdulhafiz O Adesunkanmi, Adewale O Adisa*, Samuel C Ajekwu, Olusegun K Ajenifuja, Olusegun I Alatise, Tajudeen A Badmus, Tajudeen O Mohammed, Olalekan Olasehinde, Abdulkadir A Salako, Oludayo A Sowande, Ademola O Talabi, Funmilola O Wuraola

O.L.A. Catholic Hospital, Oluyoro, Ibadan: Paul Aderemi Adegoke, Abidemi Akinloye, Ayodeji Akinniyi, Joseph Ejimogu, Ideyonbe Samuel Eseile, Olakayode Olaolu Ogundoyin*, Amos Okedare, Dare Isaac Olulana, Omolara Omotola, Francis Sanwo

Uniosun Teaching Hospital: Collins C Adumah, Adewale O Ajagbe, Olugbenga P Akintunde, Opeyemi Q Asafa, Kehinde Awodele, Amogu K Eziyi, Adeniyi O Fasanu, Olufemi O Ojewuyi*, Abiodun R Ojewuyi, Abisola E Oyedele, Oluwaseun A Taiwo

University of Abuja Teaching Hospital: Habiba I Abdullahi, Nathaniel D Adewole, Teddy E Agida, Eunice E Ailunia, Oseremen Aisuodionoe-Shadrach, Godwin O Akaba, Janet Alfred, Terkaa Atim, Kehinde G Bawa, John Y Chinda, Esther B Daluk, Sefiu B Eniola, Augusta O Ezenwa, Stephen E Garba, Ndubuisi Mbajiekwe, Philip M Mshelbwala, Ngozi O Ndukwe, Idoko P Ogolekwu, Alexander A Ohemu, Samson Olori*, Olabisi O Osagie, Samuel A Sani, Salisu Suleiman, Helen Sunday, Nancy O Tabuanu, Aminu M Umar

University of Benin Teaching Hospital: Peter I Agbonrofo*, Alexander I Arekhandia, Morrison E Edena, Raymond A Eghonghon, Joel E Enaholo, Genesis Ida, Stanley N Ideh, Oseihie I Iribhogbe, Omorodion O Irowa, Maradona E Isikhuemen, Oluwatomi R Odutola, Kester O Okoduwa, Scott O Omorogbe, David Oruade, Osasumwen T Osagie, Osarenkhoe Osemwegie

University College Hospital, Ibadan: Rukiyat A Abdus-Salam, Sikiru Adekola Adebayo, Oluwasanmi A Ajagbe, Akinlabi E Ajao, Gboyega Ajibola, Omobolaji O Ayandipo, Kelvin I Egbuchulem, Hyginus O Ekwuazi, Peter Elemile, Adegbolahan Fakoya, Oluwasegun C Idowu, David O Irabor, Taiwo A Lawal*, Olatunji O Lawal, Olakayode O Ogundoyin, Oluwabukade Ojediran, Naomi Olagunju, Akinsola T Sanusi, Augustine O Takure

University of Ilorin Teaching Hospital: Lukman Olajide Abdur-Rahman*, Mary Oluwadamilola Adebisi, Nurudeen Abiola Adeleke, Rafiat Tinuola Afolabi, Isiaka Ishola Aremu, Jibril Oyekunle Bello, Robiat Bello, Abdulwahab Lawal, Saheed Abolade Lawal, Adeolu Ojajuni, Sabur Oyewale, Hadijat Olaide Raji, Olayinka Sayomi, Asimiyu Shittu

University of Port Harcourt Teaching Hospital: Victor Abhulimen, Patrick O Igwe, Ikechukwu Enyinnaya Iweha, Raphael E John, Nnyonno Okoi, Philemon E Okoro*, Vaduneme Kingsley Oriji, Ibiene T Oweredaba

Rwanda

Bushenge Provincial Hospital: Japhet Mizero, Immaculee Mutimamwiza, Francoise Nirere, Irenee Niyongombwa*

Butaro Hospital: Jean Paul Majyabere*

Byumba Hospital: Anastase Byaruhanga, Rongin Dukuzimana, Jean Aimable Habiyakare*, Marie Gloriose Nabada, Marcel Uwizeye

Kabgayi Hospital: Mathias Ruhosha*

Kibagabaga District Hospital: joselyne Igiraneza, Faustine Ingabire, Aloys Karekezi, Jean pierre Masengesho, Christophe Mpirimbanyi*, Lydia Mukamazera, Clemence Mukangabo, Jean Paul Niyomuremyi, Gabriel Ntwari, Celestin Seneza, Divine Umuhoza

Kibogora Hospital: Sosthene Habumuremyi, Alphonsine Imanishimwe, Salathiel Kanyarukiko, Francine Mukaneza, Deborah Mukantibaziyaremye, Aphrodis Munyaneza, Gibert Ndegamiye, Pierrine Nyirangeri, Ronald Tubasiime*, Jean Claude Uwimana

Kibungo Referral Hospital: Moses Dusabe, Emelyne Izabiriza, Hope Lydia Maniraguha, Christophe Mpirimbanyi*, Josiane Mutuyimana, Olivier Mwenedata, Elisee Rwagahirima, Job Zirikana

Kibuye Referral Hospital: Isaie Sibomana*

King Faisal Hospital: Desire Rubanguka*, Josine Umuhoza, Roda Uwayezu, Leoncie Uzikwambara

Ruhengeri Referral Hospital: Aime Dieudonne Hirwa*, Elysee Kabanda, Salomee Mbonimpaye, Christine Mukakomite, Piolette Muroruhirwe

Rwamagana Provincial Hospital: Herbert Butana*, Moise Dusabeyezu, Athanasie Mukasine, Jean N Utumatwishima

Rwanda University Teaching Hospital of Kigali (Hub): Mediatrice Batangana, Georges Bucyibaruta, Sosthene Habumuremyi, Jean de Dieu Haragirimana, Alphonsine Imanishimwe, Allen J C Ingabire, Violette Mukanyange, Emmanuel Munyaneza, Emmanuel Mutabazi*, Espoir Mwungura, Isaie Ncogoza, Faustin Ntirenganya*, Jeannette Nyirahabimana, Dancilla Nyirasebura, Christian Jean Urimubabo

University Hospital of Butare: Anaclet Dusabimana, Sam Kanyesigye, Robert Munyaneza*, Jean Yves Shyirakera

South Africa

Chris Hani Baragwaneth (Hub): Maria Fourtounas, Mary Augusta Adams, Chikwendu Jeffrey Ede, Gabriella Hyman, Mpho Nosipho Mathe, Rachel Moore*, Ncamsile Anthea Nhlabathi, Hlengiwe Samkelisiwe Nxumalo, Nnosa Sentholang, Mmule Evelyn Sethoana, Paul Wondoh

Helen Joseph Hospital: Zain Ally*, Aimee Domingo, Philip Munda, Chido Nyatsambo, Victor Ojo, Rudo Pswarayi,
